# Distinguishing actual from 3D-printed bite marks in forensic odontology: accuracy and reliability of digital analysis

**DOI:** 10.1007/s00414-025-03712-x

**Published:** 2026-01-21

**Authors:** Uğur Kayhan, Safa Özden, Mahmut Şerif Yıldırım

**Affiliations:** 1 Department of Forensic Medicine, Faculty of Medicine, Afyonkarahisar Health Sciences University, Afyonkarahisar, Turkey; 2 Department of Prosthodontics, Faculty of Dentistry, Afyonkarahisar Health Sciences University, Afyonkarahisar, Turkey; 3 Department of Forensic Medicine, Faculty of Medicine, Uşak University, Uşak, Turkey

**Keywords:** Bitemark misidentification, Forensic science, Forensic odontology, 3D dental modelling, Evidence falsification

## Abstract

The convenience of gathering evidence is enhanced by three-dimensional (3D) scanner and printer technologies, however they also raise the risk of creating false evidence. The purpose of this study is to analyze the accuracy of bite marks obtained from digital dental models using quantitative methodologies, as well as to investigate the potential for bite marks obtained from models manufactured using 3D printing technology to produce false evidence. Between the ages of 18 and 25, 15 volunteers who had not undergone orthodontic treatment and had a class I skeletal relationship were included in the study and maxillary impressions were taken with an intraoral scanner (IOS). Maxillary models were produced with a 3D printer from digital impressions. Using dental silicone and dental wax materials often used by forensic odontologists, bite marks were obtained from both volunteers and their 3D models. Similarity analysis of bite marks was performed using the superimposition method using a 3D surface matching software program (Geomagic Control X 2020; 3D System). Following superimposition, RMS (Root Mean Square) values were calculated and utilized for comparison. The bite marks from participants differed significantly from those from 3D printed models (*P* < .05). While bite marks obtained from 3D printed models first looked to be identical to the actual marks, digital scanning and superimposition analysis revealed significant variances. Advancements in 3D printing technology, as well as their ease of use, may enhance the possibility of evidence fabrication. While this study demonstrates that there are discernible distinctions between actual bite marks and artificials, consistent techniques are required to improve the trustworthiness of digital data in forensic odontology.

**Supplementary Information:**

The online version contains supplementary material available at 10.1007/s00414-025-03712-x.

## Highlights

• Effect size (Cohen’s d) and RMS-based analysis demonstrated a clinically meaningful difference between original and 3D-printed bite marks.

• 3D scanning and printing errors were quantitatively assessed and found to be negligible for forensic applications.

• The study introduced ROC analysis as an objective method to determine threshold values for distinguishing artificial bite marks.

• Findings support the integration of digital bite mark analysis into standardized forensic odontology protocols to improve fraud detection.

**Supplementary Information:**

The online version contains supplementary material available at 10.1007/s00414-025-03712-x.

## Introductıon

Forensic odontology, which is a component of forensic sciences, is described as the study of dentistry that contributes to legal processes [1]. Findings from teeth and tooth marks are assessed odontologically and contribute to forensic investigations in a variety of areas, including forensic medical identification, determining abuse and the abuser in child abuse instances, bite mark analysis, and age determination [2].

Teeth can be used as a weapon to damage or as a tool for sexual assault, and the use of bite marks to identify the perpetrator has a long history in forensic research. Bite marks were first used in forensics in 1870 as evidence in a murder case. In the incident, a one-on-one match was made between the bite marks found on the victim’s body and the tooth structure of the suspect, thus contributing to the clarification of the crime [3]. The acceptance of bite marks as a systematic form of forensic evidence and their connection to standard procedures was mainly in the United States [4]. Throughout this process, many procedures and materials have been developed to compare suspected dental structures to crime scene results. To ensure the most precise record of the 3D structure of tooth marks, molding materials including dental silicone and wax, as well as bite-sized food items like chocolate, cheese, and fruits like apples are used [5]. Such surfaces play a significant role in the forensic process, both by preserving the details of bite marks and allowing analysis of individual tooth morphology [6–8].

The contamination of the bite mark with saliva allows DNA analysis. Thus, cases in which bite marks were observed but no definitive proof was established before the discovery of DNA fingerprints were re-examined. DNA analysis of materials retrieved from crime scenes, in cases where bite marks were used to make decisions has been shown to be erroneous. This has led to the conclusion that bite marks may be invalid, untrustworthy, or defective [4, 9] .

3D technology has made significant contributions to the field of forensic dentistry, particularly in the analysis of intraoral scans (IOS) and bite mark analysis [10]. During the COVID-19 global pandemic, the use of digital scanning devices grew dramatically to limit the danger of infection [11]. These methods have played a significant role in providing a safer and more efficient evidence collection process by minimizing physical contact. Preliminary studies have demonstrated the potential of IOSs to facilitate quantitative comparisons between dental models and bite marks [12, 13]. In contrast to traditional visual comparisons, this method delivers a more objective and reliable examination [14]. Furthermore, it was found that the accuracy of 3D models obtained using monoscopic photogrammetry techniques with smartphone cameras was statistically similar to those obtained with professional intraoral scanners [10]. This finding suggests that monoscopic photogrammetry techniques with smartphone cameras can be used as a low-cost, accessible alternative. It has been stated that these developments have improved the quality of evidence in forensic cases and significantly improved the efficiency of the process by facilitating digital data sharing between experts [15].

This study is designed based on the assumption that dental models obtained with IOS and 3D printing can reliably and accurately replicate individuals’ actual bite marks. The aim is to see if quantitative analysis of data gathered by digitally recording bite marks using recent technological improvements could distinguish between artificial and actual bite marks. The purpose of this study is to establish methods for assessing the reliability of digital analyses and preventing potential errors in the case of artificial bite marks generated using 3D printing technology. The null hypothesis was that there would be no difference between actual bite marks and artificial bite marks generated with 3D printed models on dental silicone and dental wax materials.

## Materıals and methods

### Experimental groups

A total of 15 volunteers (7 females and 8 males), aged 18–25 years, were recruited for this study. Sample size adequacy was determined using G*Power software with a two-tailed t-test, assuming an effect size of 0.5, α = 0.05, and 1 − β = 0.80. This calculation indicated that a minimum of 37 samples would be required to achieve 80% statistical power; however, based on previous pilot data and the exploration nature of this study, a sample size of 15 was selected [16]. The inclusion criteria were no previous orthodontic treatment and Class I skeletal and dental relationships. Individuals with severe incisor crowding or significant dental anomalies were excluded from the study to minimize confounding variables. This study did not consider differences in jaw size and arch width between individuals.

### Model production

**Fig. 1:**
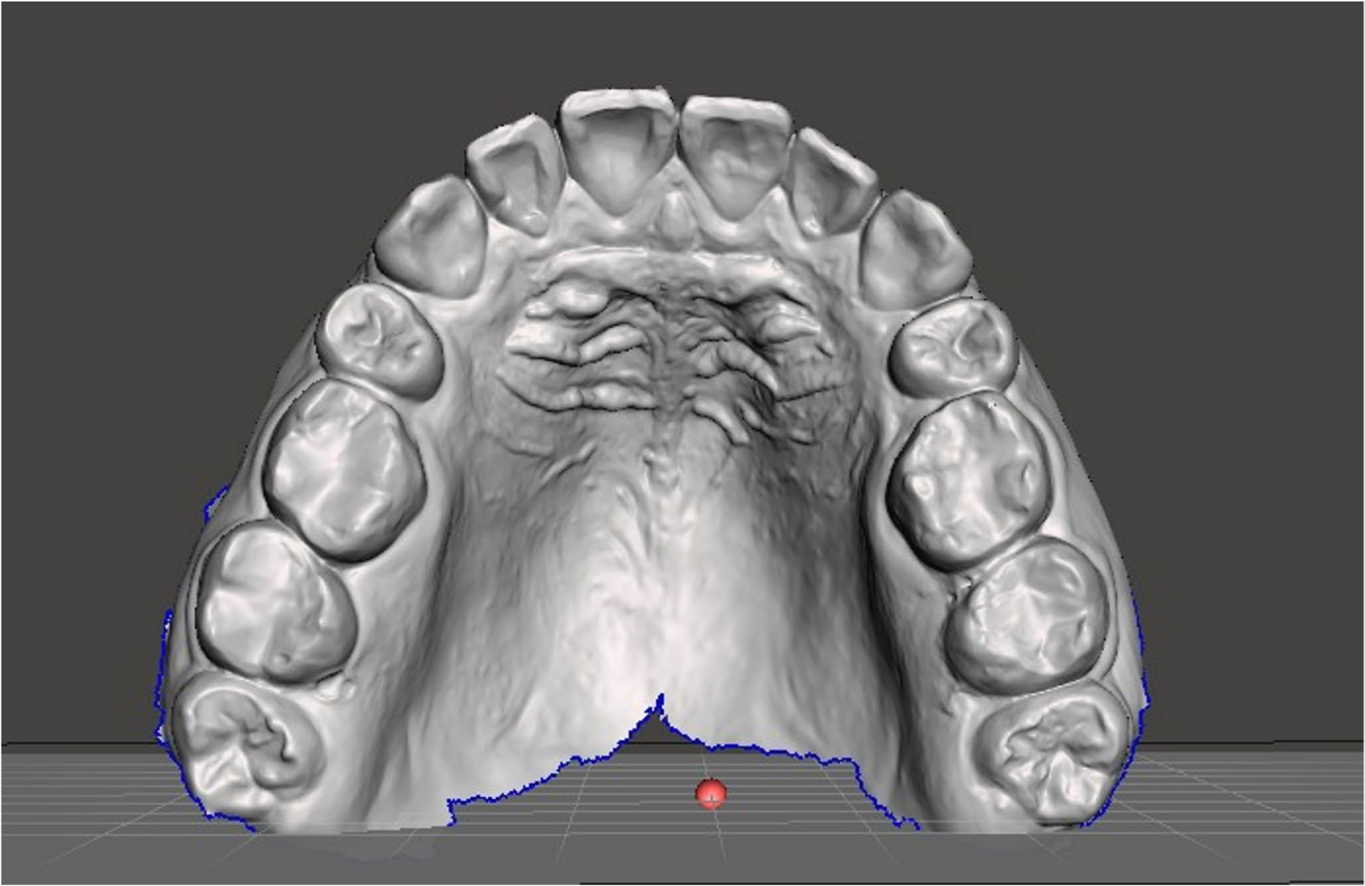
Occlusal view of maxillary model obtained with 3D scanner

The maxillary impression of each volunteer was recorded in “.ply” format by the same operator using an intraoral scanner (Trios 3; 3Shape, Denmark) under ambient light of approximately 1000 lx (Fig. 1). Unnecessary soft tissue and tooth areas were removed using mesh manipulation software (Meshmixer; Autodesk). The digital files were processed with slicing software (Chitubox V1.7.0; Chitubox) using a layer thickness of 50 μm and a printing angle of 45° [17]. A high-resolution resin 3D printer (Phrozen Sonic Mini 8 K DMSL; Phrozen) was used. The models were printed using model resin (Alias model resin; Dokuz Kimya), washed with isopropyl alcohol (99%) twice for 3 min, and cured to finalize production. Each volunteer and their corresponding model were numbered and matched.

### Obtaining bite marks

Condensation silicone putty (Zetaplus; Zhermack) and three layers of dental modelling wax (Cerewax; PD Company) were used to record bite marks from both volunteers and their 3D models. The maxillary canine-to-canine region was standardized as the reference area for all bite mark registrations. All bite marks were recorded under controlled conditions by the same operator to minimize inter-operator variability. Volunteers were instructed to bite with moderate force until full occlusal contact was achieved, and the bite was maintained for approximately 3 s to ensure reproducible impressions. For 3D-printed models, a mechanical press was used to apply a consistent biting force during the creation of bite marks, simulating natural occlusal pressure.

**Fig. 2:**
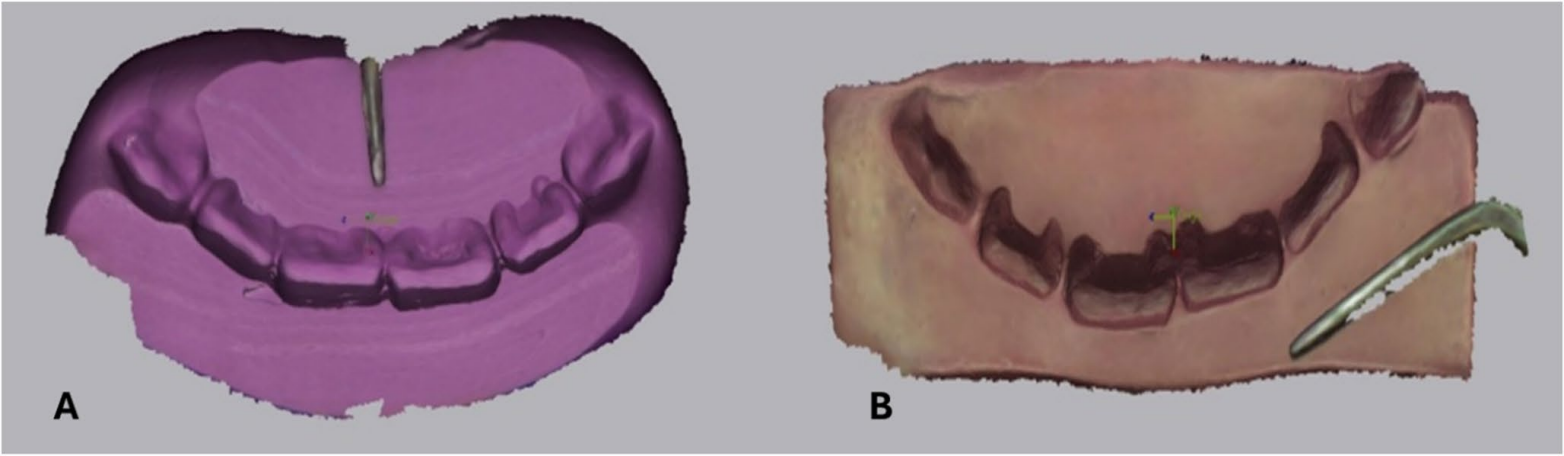
Digital PLY files of bite marks (A: dental silicone, B: dental wax)

To reduce potential deformation of materials, impressions were stored at room temperature and analyzed within one hour of registration. After 24 h, bite mark recording was repeated using the same procedure to assess reproducibility and calculate the intercorrelation coefficient. Each bite mark was then labeled and digitally archived for subsequent surface matching analysis (Fig. 2).

### 3D comparison

Surface matching analysis was conducted using metrology-grade software (Geomagic Control X 2020; 3D Systems) to allow bite mark comparison. With the use of a colorimetric scale, this software determines the point distance differences between two surfaces and graphically illustrates the positive and negative deviations between them. The Root Mean Square (RMS) value, which quantifies the surface deviation between two bite marks, was calculated using the following formula:

$$\:RMS=\surd\:\left[{{\Sigma\:}}_{i}^{=1n}{\left({X}_{1,i}-{X}_{\mathrm{2,1}}\right)}^{2}/n\right]$$

**Fig. 3:**
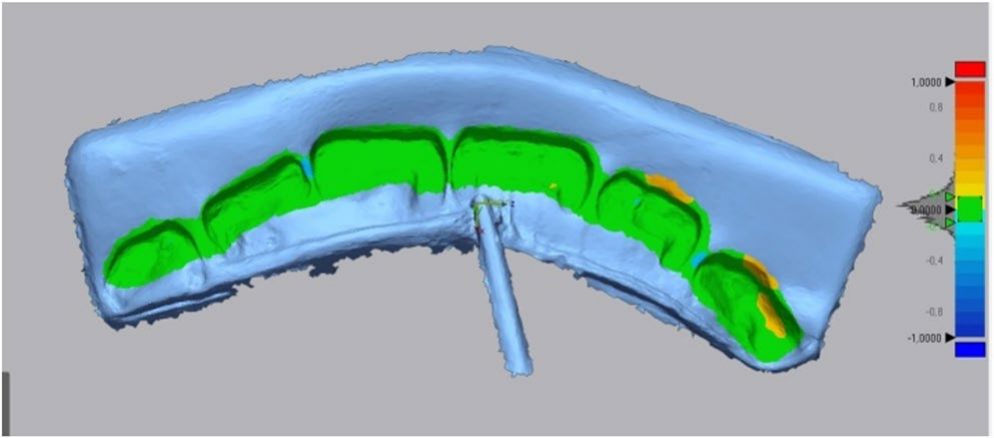
Superimposition on wax with color map diagram

Through this formula, similarities or differences between bite marks are quantitatively evaluated. In the formula, ‘X₁,i’ represents the data point ‘i.’ on the reference surface, and ‘X₂,i’ represents the corresponding data point ‘i.’ on the test group surface; ‘n’ refers to the total number of points compared. In order to make comparisons, the initial alignment was performed by determining at least three anatomical reference points such as the cusp tip of the canine teeth and the mesioincisal angles of the central teeth on the 3D images. The software’s ‘Best-Fit Alignment’ tab was then used to create the best surface alignment possible. As a result of the alignment process, a colorimetric color scale coded in green (0 micron), red (+ 100 micron) and blue (− 100 micron) tones and corresponding RMS values were obtained (Fig. 3).

**Fig. 4:**
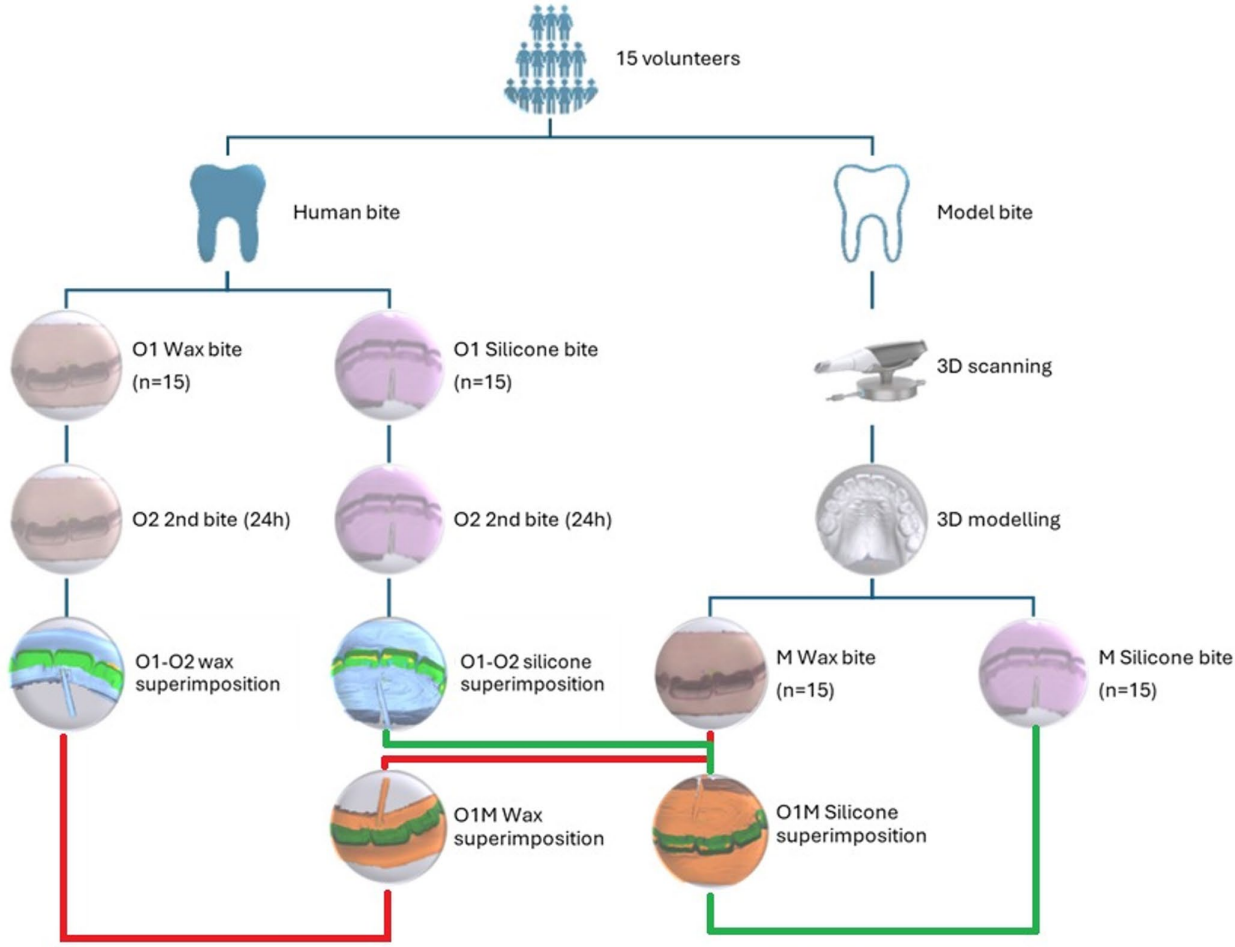
Basic workflow chart used in the experimental model, along with a summary of the comparisons made

The initial actual bite marks (O1) acquired from participants were matched with the bite marks (M) obtained from 3D printed models using superimposition software, and RMS values were computed. Furthermore, the second actual bite marks (O2) taken 24 h later under similar conditions to O1 were matched with their own pairs (wax or silicone), and RMS values were obtained (Fig. 4). These values, calculated separately for dental wax and dental silicone materials, were statistically compared. The distribution of the data was analyzed using the Shapiro-Wilk normality test, and it was found to be normal. As a result, group comparisons were made using the Independent Samples t-test, and analyses were conducted using statistics software (SPSS v24.0; IBM Corp.).

### Statistical analysis

The distribution of the data was evaluated using the Shapiro–Wilk test. Since the data showed normal distribution, group comparisons were performed using independent samples t-tests. Bonferroni correction was applied to control for multiple comparisons. A mixed-effects model was also used to account for repeated measurements within subjects and to analyze the effects of material type (dental wax and dental silicone) and bite mark origin (actual or model-generated).

### Effect size analysis

In addition to P-values, effect sizes (Cohen’s d) were calculated to assess the practical significance of the differences between actual and 3D model bite marks. Cohen’s d values were interpreted as small (0.2), medium (0.5), or large (0.8) Cohen’s d was computed for each material (dental wax and dental silicone) using the following formula:

$$\:d=\frac{{\mathrm{Mean}}_{{O}_{1}M}-{\mathrm{Mean}}_{{O}_{1}{O}_{2}}}{\sqrt{\frac{{\mathrm{SD}}_{{O}_{1}M}^{2}+{\mathrm{SD}}_{{O}_{1}{O}_{2}}^{2}}{2}}}$$

where O1M represents the RMS values of the original bite marks relative to the model bite marks, and O1O2 represents the RMS values of the first and second original bite marks.

### ROC analysis

Receiver Operating Characteristic (ROC) analysis was performed to evaluate the discriminatory power of RMS values in distinguishing actual bite marks from 3D-printed model bite marks. The area under the curve (AUC) was used to quantify classification accuracy, and the optimal cut-off value was determined using the Youden Index.

### Ethical approval

The methods and aims of the study were reviewed by Afyonkarahisar Health Sciences University Non-Interventional Research Local Ethics Committee and approved with the decision number 2022/433 on 02/09/2022.

## Results

### Accuracy of the method

#### Correlation coefficient test

Bite marks taken from volunteers 24 h apart were compared to assess the method’s accuracy and repeatability. The mean RMS values of bite marks made on dental wax and dental silicone materials did not differ significantly (*P* > .05). The method’s great reliability and reproducibility are demonstrated by the results of the superimposition analysis of the data using these two traditional materials, which are commonly used by forensic odontologists (Table 1).

**Table 1:** Correlation analysis of RMS values from superimposed actual bite marks

Measurements	O_1_O_2_ (Wax)	O_1_O_2_ (Dental silicone)	p
Mean RMS Values	0.081 ± 0.12 mm	0.070 ± 0.21 mm	0.631

O1O2: First actual bite mark and second actual bite mark superimposition

#### 3D printer sensitivity

In the analysis conducted to evaluate the impact of 3D printer related production errors and the digital workflow process on the study, the STL (Standard Tessellation Language) file obtained with an IOS of a participant’s upper jaw was used as a reference. 15 models were produced from this STL file, and the produced models were scanned again with an IOS. The digital data obtained were overlapped with each other by the superimposition method. As a result of statistical analysis, it was determined that the mean difference was not clinically significant (0.0056 ± 0.0004 mm). Similarly, repeated scanning of the same model revealed a negligible mean difference (0.0034 ± 0.0008 mm). These findings indicate that error factors related to both model production and scanning were effectively minimized, supporting the accuracy of the digital workflow.

#### Scanner sensitivity

In order to determine the effect of scanner related errors in the study, a single model produced from the original STL file was scanned 15 times repeatedly in order to evaluate the impression deviation due to the IOS after standardizing environmental factors such as ambient light. After comparing the acquired scan data, it was concluded that the mean difference (0.0034 ± 0.0008 mm) was not significant enough to influence the study’s findings. This result demonstrates that intraoral scanning error factors are successfully managed.

### Superimposition of model bite marks with actual bite marks

The RMS values of the actual bite marks were 0.081 ± 0.12 mm for dental wax and 0.070 ± 0.21 mm for dental silicone in the analyses undertaken to assess the detectable difference between the bite marks and the artificial bite marks created using the models. When the actual bite marks and artificial model bite marks were superimposed, the mean RMS for the dental wax group was 0.182 ± 0.26 mm, whereas the mean RMS for the dental silicone group was 0.180 ± 0.16 mm. The bite marks derived from the model were found to differ from the actual bite marks in a statistically significant way (*P* < .05; Table 2).

**Table 2:** Mean RMS values of actual and artificial bite marks

Material	O_1_O_2_ (mm)	O_1_M (mm)	*p*
Dental wax	0.081 ± 0.12	0.182 ± 0.26	< 0.002^*^
Dental silicone	0.070 ± 0.21	0.180 ± 0.16	< 0.001^*^

*P < .05

O1O2: First actual bite mark and second actual bite mark superimposition

O1M: First actual bite mark and model bite mark superimposition

Effect size analysis revealed moderate effect sizes for both dental wax (d ≈ 0.50) and dental silicone (d ≈ 0.55), supporting the practical significance of the differences observed between the original and 3D model bite marks.

Individual RMS values for each participant and control samples are presented in Supplement 1. These data illustrate both inter‑individual variation and baseline values from control groups.

## Discussion

Bite mark analysis is a key finding in forensic crime scene reconstruction. While analytical techniques are evolving in conjunction with technological improvements, these same technological instruments also carry the risk of criminals employing them to falsify evidence. Based on our findings, we rejected the null hypothesis that there would be no difference between actual bite marks and artificial bite marks made with 3D-printed models, dental silicone, and dental wax materials.

The study’s inclusion criteria included not having received orthodontic treatment and being free of dental anomalies. Individuals getting orthodontic treatment have conventional tooth sequences similar to the ideal, which decreases differences between people and reduces the authenticity of bite marks [17]. Patients with tooth alignment disorder, missing teeth or dental anomalies at a level that facilitates detectability were not included in the study [17]. As such scenarios can result in impression mistakes or discrepancies in bite mark analysis, reducing the dependability of the data. To improve the study’s methodological consistency and data accuracy, healthy individuals with complete tooth alignment were chosen. IOS and 3D printer systems, which are increasingly used in odontology, provide advantages in the detection of bite marks [18, 19]. These technologies offer significant convenience in forensic investigations by making it possible to obtain digital data with high accuracy and to produce physical models quickly and precisely [13–22]. When compared to conventional materials, the reproducibility of digital models and the lower danger of data loss give a key benefit in terms of evidence integrity. However, the growing use of 3D printer and scanner technology introduces new challenges in forensic tasks, including the creation of false evidence. It is critical to develop methods for detecting fake bite marks created using printed models, as well as to call into question the reliability of existing techniques. According to the findings of this study, IOS and 3D printer systems have the potential to be used in forensic odontology for evidence collection and prevention of falsification.

Dental wax and dental silicone materials, which are frequently used in the literature for reasons related to material quality and accessibility of supply, as well as providing reproducibility and standardization of the studies, were selected for creating bite marks [23]. When collecting bite mark evidence, material selection is essential to the quality and accuracy of the markings. While the literature describes the use of food items such as chocolate or cheese for bite mark collecting in practical applications [8], the inclusion of such organic ingredients can have a negative impact on the reliability of evidence. Chocolate and cheese are structurally inhomogeneous, meltable, and prone to deformation. This might result in decreased bite mark clarity, missing detail, and measuring mistakes. Furthermore, the rapid growth of microorganisms in these materials complicates evidence preservation and makes them unsuitable for long-term storage and analysis. In forensic odontology applications, bite mark evidence must be produced on objective, reproducible, and long-lasting materials. As a result, it is critical to prefer dental silicone, dental wax, or similar standard materials over foods including chocolate and cheese in terms of evidence accuracy and admissibility in court.

In recent years, a large number of studies have appeared in the literature on erroneous results related to bite mark analysis [4, 9]. However, technological advances necessitate a rethinking of the current role of bite mark analysis in forensic medicine. In this context, rapid technical advancements have had a substantial impact on bite mark analysis. To improve the repeatability and reliability of the information gathered, printer and scanner accuracies were examined using the intercorrelation coefficient, hence reducing the error sources connected with these parameters. This approach is also emphasized in Fournier et al.‘s [13] study on the detection of bite marks using 3D scanners, as methodological errors can lead to misinterpretations about the reliability of digital systems. The use of digital technologies in the field of forensic odontology provides more sensitive and objective data compared to traditional methods. Intraoral scanners provide digital images close to real colors with high accuracy and color scanning in the field of dentistry. Today, it has been reported in the literature that 3D intraoral scanning can be successfully performed with some mobile phones [10]. These devices’ ease of use can improve the reliability of the results by allowing for faster recordings from the crime scene. The deployment of such devices at crime scenes where bite marks are found can improve the effectiveness of forensic investigations by speeding up the collection of physical evidence. Furthermore, direct digitization of data gathered through digital scanning methods enables the preservation of evidence integrity and more extensive examination.

The exponential development of digital technologies creates new challenges. The hazards of software and hardware mistakes or manipulation that may occur during the generation of digital evidence are particularly notable. As a result, developing standard methods is crucial for assuring the validity of digital evidence used in forensic investigations. In our study, we determined whether the hypothesized 3D models could replicate the actual bite marks and distinguish between them. The superimposition of the actual bite marks at different times (O1O2) yielded 0.081 ± 0.12 mm for dental wax and 0.070 ± 0.21 mm for dental silicone. The superimposition of the actual bite marks at different times (O1O2) yielded the same results of 0.182 ± 0.26 mm and 0.180 ± 0.16 mm for the actual bite-model (O1M). This demonstrated that, while it was previously assumed that the dental model, which was designed to reproduce the dentition predicted in our pre-study hypothesis, replicated the bite mark, this distinction could be identified via digital analysis. This highlights the possible use of digital analysis in forensic odontology.

In this study, bite marks on dental wax and dental silicone were examined in light of the literature. All volunteers were individuals with Class I dentitions who had not received orthodontic treatment. This methodology narrows the range of methods by excluding various dental variations. The idea of producing artificial bite marks was investigated in a laboratory setting using digital models. Given the complexities of the data gathered from actual crime scenes, not all complicated aspects that could influence the outcomes were considered in the research.

### Limitations

Considering that there are other materials recommended in literature (e.g., vinyl polysiloxane, acrylic, etc.) [6, 24] and unlimited materials that can be examined in real events, it is not possible to evaluate all these materials in one study. However, in the future, this limitation should be eliminated with different studies.

Another drawback of the study is that it does not cover varying instances in terms of jaw dimensions in order to standardize experiment techniques. This may constitute a potential source of error in the obtained RMS values ​​and should be taken into account in future studies. The technique used in this study does not fully represent real-world instances. The experimental approach did not account for condylar movement, and only people with Class I occlusion were studied. Furthermore, this model does not account for the heterogeneity, mobility, and surface abnormalities of skin tissue observed in real-world occurrences. In most forensic situations, bite marks are detected in two dimensions and are more difficult to assess. These constraints may restrict the findings’ direct applicability to real-world circumstances.

## Conclusions

This study shows that although 3D-printed dental models and intraoral scans may duplicate bite marks with excellent technical precision, substantial discrepancies exist between actual and artificial markings. Importantly, even under the most standardized laboratory circumstances, it was not able to exactly duplicate bite marks with 3D reconstruction. Given that bite marks appear on human skin, which is a diverse, elastic, and dynamic tissue, the credibility of bite mark evidence should be assessed with caution. While digital superimposition shows statistically significant changes, its actual application in forensic investigations is disputed. As a result, bite mark analyses should be read seriously, and future research should concentrate on skin-based models and non-Class I occlusions to better reflect real-world forensic situations.

## Supplementary Information

Below is the link to the electronic supplementary material.

Supplementary Material 1
