# Supplementary material for: Distinguishing actual from 3D-printed bite marks in forensic odontology: accuracy and reliability of digital analysis
Source: Int J Legal Med. 2026 Jan 21;140(3):1775–83. doi: 10.1007/s00414-025-03712-x (PMC13161320; doi:10.1007/s00414-025-03712-x)
Supplement: Supplementary file 1 — Supplementary Material 1 [file 414_2025_3712_MOESM1_ESM.docx]

| **Supplement 1.** Participant specific RMS values. | | |
| --- | --- | --- |
| *Participant* | *Material* | *RMS value* |
| Participant 1 | Wax | 0,1594 |
| Participant 2 | Wax | 0,1969 |
| Participant 3 | Wax | 0,1199 |
| Participant 4 | Wax | 0,0701 |
| Participant 5 | Wax | 0,0991 |
| Participant 6 | Wax | 0,1266 |
| Participant 7 | Wax | 0,4423 |
| Participant 8 | Wax | 0,3265 |
| Participant 9 | Wax | 0,1087 |
| Participant 10 | Wax | 0,1184 |
| Participant 11 | Wax | 0,2505 |
| Participant 12 | Wax | 0,2182 |
| Participant 13 | Wax | 0,1204 |
| Participant 14 | Wax | 0,1242 |
| Participant 15 | Wax | 0,1091 |
| Participant 1 | Silicone | 0,2515 |
| Participant 2 | Silicone | 0,0889 |
| Participant 3 | Silicone | 0,2114 |
| Participant 4 | Silicone | 0,1311 |
| Participant 5 | Silicone | 0,0745 |
| Participant 6 | Silicone | 0,0942 |
| Participant 7 | Silicone | 0,1497 |
| Participant 8 | Silicone | 0,1825 |
| Participant 9 | Silicone | 0,204 |
| Participant 10 | Silicone | 0,2177 |
| Participant 11 | Silicone | 0,1635 |
| Participant 12 | Silicone | 0,1463 |
| Participant 13 | Silicone | 0,2377 |
| Participant 14 | Silicone | 0,2586 |
| Participant 15 | Silicone | 0,1577 |
| Control 1 | Wax | 0,0684 |
| Control 2 | Wax | 0,0575 |
| Control 3 | Wax | 0,0603 |
| Control 4 | Wax | 0,0425 |
| Control 5 | Wax | 0,1145 |
| Control 6 | Wax | 0,0964 |
| Control 7 | Wax | 0,13 |
| Control 8 | Silicone | 0,0497 |
| Control 9 | Silicone | 0,0401 |
| Control 10 | Silicone | 0,1612 |
| Control 11 | Silicone | 0,1362 |
| Control 12 | Silicone | 0,0313 |
| Control 13 | Silicone | 0,0252 |
| Control 14 | Silicone | 0,0423 |
